# The 24-h profile of the DNA repair enzyme 8-oxoguanine glycosylase 1 (OGG1) is associated with age, TNF-α, and waist circumference in healthy adults

**DOI:** 10.1007/s11357-023-01012-z

**Published:** 2023-11-22

**Authors:** Per Arkenberg, Manuela Dittmar

**Affiliations:** Department of Human Biology, Zoological Institute, Christian-Albrechts-University, Am Botanischen Garten 9, 24118 Kiel, Germany

**Keywords:** Aging, Human, OGG1, TNF-α, Cortisol, Waist circumference

## Abstract

It is unknown how the DNA repair enzyme OGG1 relates to healthy aging in humans, in particular to inflammaging, that is associated with increased levels of TNF-α. This study aimed (1) to investigate how 24-h profiles for OGG1 change during healthy aging and (2) to analyze the relationship of OGG1 with TNF-α, central body fat, cortisol and oxidative DNA/RNA damage. In a cross-sectional study in 20 healthy older and 20 young women, salivary levels of OGG1, TNF-α, cortisol and oxidative DNA/RNA damage were quantified by ELISAs every 4 h for a 24-h period. Trunk circumferences were taken as measures of central body fat. Older women, compared to young women, exhibited significantly lower protein levels of OGG1 throughout the whole 24-h period, a 2.5 times lower 24-h mean level for OGG1 (*P* < 0.00001) and loss of 24-h variation of OGG1. Both age groups demonstrated significant 24-h variation for TNF-alpha, cortisol and oxidative damage. The 24-h mean level for TNF-α was more than twice as high in older compared to young women (*P* = 0.011). Regression analysis detected that age, TNF-α and waist circumference were negative significant predictors of OGG1, explaining 56% of variance of OGG1 (*P* < 0.00001), while levels of cortisol and oxidative damage were no predictors of OGG1. Results indicate a strong decrease of protein levels of OGG1 and a loss of 24-h variation during natural cellular aging. The negative relationship, found between OGG1 and TNF-α and between OGG1 and waist circumference, suggests involvement of proinflammatory processes in DNA repair.

## Introduction

Aging is accompanied by increased levels of oxidative stress, which induces damage to genomic and mitochondrial DNA [1]. The most prevalent oxidatively induced DNA lesion is 8-oxoguanine (8-oxoG) [2]. The DNA repair enzyme 8-oxoguanine DNA glycosylase (OGG1) removes 8-oxoG from oxidatively damaged DNA by base excision repair [3]. The enzyme OGG1 is ubiquitously expressed in human tissues [4]. Animal studies demonstrated an age-related decrease in mRNA and protein levels of OGG1 [5]. In addition, there is evidence that gene expression and enzymatic activity of OGG1 follow a circadian (24-h) pattern in human blood lymphocytes [6]. Data on 24-h variation of OGG1 are restricted to young adults, while data from older adults are still missing. Knowledge on 24-h variation of OGG1 in older adults is needed because aging is associated with alterations in 24-h rhythms [7] that may affect cellular homeostasis.

Reactive oxygen species (ROS), which cause damage to DNA, are produced not only during metabolic processes, but also in response to inflammatory processes [8, 9]. Inflammation is associated with higher circumferences of the trunk [10]. A chronic low-grade systemic inflammation has been related to natural aging, even in the absence of risk factors and diseases [11], termed inflammaging [12]. The proinflammatory cytokine tumor necrosis factor alpha (TNF-α) can be used as a biomarker for inflammaging. The inflammatory activities of TNF-α are downregulated in part by the anti-inflammatory hormone cortisol [13]. Both TNF-α and cortisol have been related to the DNA repair enzyme OGG1. Animal findings showed that TNF-α inactivates via oxidation a common *OGG1* variant [14]. A study in humans reported a positive relationship between plasma cortisol and *OGG1* gene expression in lymphocytes [6].

Animal studies showed that TNF-α also suppresses the expression of clock genes, which are involved in the generation of circadian rhythms [15]. As yet, studies on the 24-h variation of protein levels of OGG1 in healthy older humans are missing. In addition, the 24-h relationship of protein levels of OGG1 with TNF-α and with cortisol in saliva have not yet been examined in healthy older humans. Therefore, the first aim of this study was to analyse whether salivary protein levels of OGG1 exhibit 24-h variation in healthy aged and whether the 24-h profiles of older adults differ from those of young adults. A cross-sectional study design was applied to compare the 24-h patterns of OGG1 levels from older and young adults. The second aim was to explore the temporal relationships of OGG1 with TNF-α and other characters by correlation analyses and to determine predictors of OGG1 by regression analysis, including trunk circumferences. Deeper knowledge of how 24-h profiles of oxidative and inflammation markers and their relationship change during human aging may help to develop measures for promoting healthy aging.

## Methods

### Subjects

Forty healthy adult women of European ancestry were enrolled in the study. They comprised 20 young women (age 20–29 years, mean ± standard deviation, 24.25 ± 2.45 years) and 20 older women (60–82 years, 68.60 ± 5.68 years). The study was confined to women because sex differences have been shown for oxidative stress levels [16], TNF-α [17] and cortisol secretion [18]. The women were recruited through flyers, by word-of-mouth and in different clubs. Women were included in the study if they were healthy, nonsmokers, had C-reactive protein < 5.0 mg/L, BMI between 18.5 and 29.0 kg/m^2^, sleep duration between 6 and 9 h, bedtimes between 21:00 and 01:00 h and a regular sleep–wake pattern. Exclusion criteria were acute and chronic illness, cancer, diabetes, psychiatric illness, oral diseases, canker sores, bleeding gums, dental treatment within the last month as well as anti-inflammatory, sleeping, and psychiatric medication. Additional exclusion criteria were physical injury, sunburn, current diet, high-performance sport, pregnancy, shift work, and jet-lag within the last three months. Inclusion and exclusion criteria were assessed by questionnaires, medical history and laboratory analysis. 38% of the women never drank alcohol and 62% only small amounts. None of the older women received hormone replacement therapy. Seven of the young women took monophasic oral contraceptives for birth control. All participants gave written informed consent. The ethics committee at the Christian-Albrechts-University of Kiel approved the study protocol (D 535/19).

### Procedure

Data was collected from June to August 2022. Participants visited the Department of Human Biology at Kiel University two times. At the first appointment, each participant received personally oral and written instructions on the study procedure. The participants were told to follow a regular sleep–wake pattern for seven days prior to the study day to stabilize their biological rhythms. They noted bedtimes for each of the 7 days. The second appointment was on study day. The participants arrived after an overnight fast at 07:30 h at the university and completed several questionnaires. The concentrations of hs-CRP and vitamin D were measured before 08:00 h and anthropometric measurements were taken. The participants stayed in a separate room for saliva collection. During the 24-h sampling period, whole unstimulated saliva was collected at 4-h intervals at 08:00, 12:00, 16:00, 20:00, 24:00, 04:00 and 08:00 h into specific sampling devices (SaliCaps, IBL, Hamburg, Germany). Saliva was collected from 08:00 to 20:00 h by one of the authors (P.A.) and from 24:00 to 08:00 h by the participants themselves, who were carefully trained and equipped with collecting devices. Within one hour before each saliva sampling, the participants refrained from brushing teeth, drinking, eating and chewing gum. Saliva samples were cleared by centrifugation at 1000 rpm for 15 min to pellet bacteria, food debris and cells. The supernatant was immediately stored in aliquots at -80 °C until analysis. The aliquots were used for determining concentrations of OGG1, DNA/RNA oxidative damage, TNF-α and cortisol. Core body temperature was measured twice at the same time-points. During the study day, the participants were not allowed to do sports, nap, drink alcohol and caffeinated drinks. All participants had breakfast, lunch and dinner, respectively, between 08:30–10:00 h, 12:30–14:00 h and 18:00–19:00 h. From 19:00 h until bedtime, no meals were allowed. From 16:00 h until bedtime, the participants wore blue light blocking glasses (400–500 nm, Prisma blue-light protect Amber PRO, Innovative eyewear, Weilheim, Germany), because blue light exposure inhibits endogenous melatonin secretion, which is important for generating robust circadian 24-h rhythms [19].

### Anthropometry, bioelectrical impedance analysis, questionnaires

Body weight, body height, body circumferences (waist circumference, abdomen circumference, hip circumference), and body composition, respectively, were determined using an electronic scale (TGF 302H, Rossmann, Burgwedel), a wall-mounted measuring device (Seca 206, Seca, Hamburg), a measuring tape, and a whole-body tetrapolar bioelectrical impedance analyzer (Nutriguard-M, Data Input, Pöcking, Germany). Body mass index (BMI) was calculated as body weight (kg) divided by the square of body height (m^2^). Morningness-eveningness preference, sleep quality and daytime sleepiness, respectively, was estimated using the Morningness-Eveningness-Questionnaire ([20], German validated version by [21]), the Pittsburgh Sleep Quality Index [22], and the Epworth Sleepiness Scale [23].

### Measurement of hs-CRP and vitamin D

The C-reactive protein (CRP) is an acute-phase protein, whose concentration increases in blood in response to inflammation. CRP was quantified to rule out the presence of acute or chronic inflammation in the body of the participants. Any participant having CRP > 5.0 mg/L was excluded from the study. High-sensitive CRP (hs-CRP) was quantified in capillary blood of the finger using the hs-CRP assay on Eurolyser CUBE-S laboratory photometer (Hitado, Dreihausen, Germany). This quick test is controlled for hematocrit. The assay range is 0.8–20 mg/L blood.

Vitamin D was quantified because a deficiency may increase levels of OGG1, DNA damage and TNF-α [24]. Vitamin D was measured using the “Vitamin D quantitative test” assay on VHC reader (Hitado, Dreihausen, Germany). This immunochromatography-based one step quick test quantifies the total 25-hydroxy vitamin D (25-OH vitamin D) in finger capillary blood. It can be used for screening for vitamin D deficiency. The assay range was 4–100 ng/ml and its sensitivity was 3.3 ng/ml.

### Oxoguanine glycosylase (OGG1) assay

The protein level of OGG1 was quantified in human saliva samples that were collected at seven time-points (08, 12, 16, 20, 24, 04, and 08 h). The OGG1 concentrations were measured using a human enzyme-linked immunosorbent assay (ELISA kit ELK3235, ELK Biotechnology, Wuhan, Hubei, China), as described by the manufacturer. The test principle is based upon a quantitative sandwich enzyme immunoassay technique. Streptavidin-HRP binds to biotinylated antibodies and provides enzyme activity for detection with a substrate system. The reaction between enzyme (HRP) and substrate (TMB) resulted in colour change. The optical density was measured with a microtiter plate reader (Multiskan FC, ThermoScientific, Darmstadt, Germany) at 450 nm. The concentrations of the samples were determined from a standard curve with seven standards, adapted to the software of the microtiter plate reader. All samples were analyzed in duplicate. The assay range was 0,16–10,00 ng/ml and the sensitivity of the assay was 0.058 ng/ml.

### Oxidative DNA/RNA damage assay

The level of oxidative damage to DNA/RNA was measured in human saliva samples that were collected at seven time-points (08, 12, 16, 20, 24, 04 and 08 h) and stored at -80 °C. A competitive enzyme-linked immunosorbent assay (ELISA kit no. 589320, Cayman Chemical, Ann Arbor, MI, USA) was used to quantify all three oxidized guanine species from DNA and RNA (8-hydroxy-2’-deoxyguanosine, 8-OHdG; 8-hydroxyguanosine, 8-OHG; 8-oxoguanine, 8-OxoG), following the manufacturer’s instructions. This quantitative assay is based on the competition between the oxidatively damaged guanine species and an 8-OH-dG-acetylcholinesterase conjugate for an DNA/RNA oxidative damage monoclonal antibody. An enzymatic reaction using Ellman’s reagent lead to a yellow product whose optical density was measured with a microtiter plate reader (Multiskan FC, ThermoScientific, Darmstadt, Germany) at 405 nm. Sample concentrations were calculated by means of the manufacturer’s table in Microsoft Excel 2016. The sensitivity of the assay approximated 30 pg/ml.

### Tumor necrosis factor alpha (TNF-α) high-sensitivity assay

The protein level of TNF-α was determined in human saliva samples that were collected at seven time-points (08, 12, 16, 20, 24, 04, and 08 h). The TNF-α concentration was measured using the high-sensitivity human Quantikine™ enzyme-linked immunosorbent assay (ELISA kit HSTA00E, R&D Systems, Minneapolis, USA), according to the manufacturer’s instructions. The test principle of the assay employs a quantitative sandwich enzyme immunoassay technique using a monoclonal antibody specific for human TNF-α, coated on a 96-well plate. After washing of the wells, a polyclonal antibody specific for TNF-α, was added to the plate. After adding enzyme-linked streptavidin and substrate solution, a colour reaction developed, which was proportional to the amount of TNF-α in the sample. Optical density was read at 570 nm and a reference wavelength of 620 nm with a microtiter plate reader (Multiskan FC, ThermoScientific, Darmstadt, Germany). The concentration of TNF-α was determined from a standard curve, which was created using the software of the microtiter plate reader. All samples were analyzed in duplicate. The sensitivity of the assay approximated 0.022 pg/ml.

### Cortisol assay

The concentration of cortisol was determined in human saliva samples, which were collected at seven time-points (08, 12, 16, 20, 24, 04, and 08 h). After screening for blood contamination, concentrations of free cortisol in saliva were measured with a quantitative enzyme-linked immunosorbent assay (ELISA kit RE52611, IBL, Hamburg, Germany), following the manufacturer’s instructions. The test principle is based upon the competition between the unknown amount of cortisol in the sample and a fixed amount of enzyme-labelled cortisol for antibodies coated onto a 96-well plate. The cortisol concentration is inversely proportional to the intensity of color change. Optical density was measured at 450 nm with a reference wavelength of 620 nm using a microtiter plate reader (Multiskan FC, ThermoScientific, Darmstadt, Germany). The cortisol concentrations were calculated from a standard curve with the software of the microtiter plate reader. The assay range was 0.005–3.00 µg/dl.

### Determination of core body temperature

Core body temperature (CBT) was measured in the right posterior sublingual pocket of the mouth with a digital thermometer (Thermoval, Hartmann, Heidenheim, Germany) at 08, 12, 16, 20, 24, 04 and 08 h. Temperature measurements were done in duplicate and the mean value was used for analysis.

### Statistical analyses

Statistical analyses were performed by means of IBM SPSS software for MS Windows, release 29.0 (IBM, Armonk, NY, USA). Data are shown as mean ± standard deviation (SD) or standard error of the mean (SEM). Outliers beyond three standard deviations from the mean were excluded from data analysis following Snedecor and Cochran [25]. Normal distribution of data was analyzed by Shapiro–Wilk test. Differences between age groups were tested using the two-tailed Student’s *t* test or Mann–Whitney-*U*-test, depending on normal distribution of data. Friedman tests were applied to test for 24-h variation of levels for OGG1, oxidative DNA/RNA damage, TNF-alpha and cortisol, separately for young and older women. Differences between day levels and night levels of the circadian parameters were compared using *t* tests for dependent samples or Wilcoxon tests, were appropriate. Mixed linear models were applied to test the interaction between time (day vs. night) and age (young vs. older women). The dependent variables were the levels for OGG1, oxidative DNA/RNA damage, TNF-alpha and cortisol as well as CBT. Log-transformed values were used for OGG1, TNF-alpha and cortisol. The fixed factors were time and age. The amplitude of the circadian cycle of CBT was calculated as amplitude percent mean (A%Mean) as follows: highest CBT value of the 24-h day minus lowest CBT value of the 24-h day, divided by the mean CBT value of the 24-h day and multiplied with 100 [26]. Pearson or Spearman rank correlation coefficients were used to explore relationships between variables, where appropriate. Multiple regression analysis was applied to examine the influence of age, oxidative DNA/RNA damage, TNF-α, cortisol and anthropometric measures (independent variables) on the 24-h mean level of OGG1 (dependent variable). Log-transformed values for OGG1, oxidative DNA/RNA damage, TNF-alpha and cortisol levels were used in the regression analysis. A two-sided *P* value of < 0.050 was considered statistically significant.

## Results

### General characteristics of study participants

Table 1 summarizes anthropometric and other characteristics of the study participants. There were no significant differences between young and older women for sleep quality, daytime sleepiness, morningness-eveningness score and BMI. By contrast, older women had a significantly higher waist circumference, higher abdomen circumference and lower body cell mass than young women. A comparison between older women aged 60–68 years vs. 70–82 years showed no significant differences for BMI (23.9 vs. 23.3 kg/m^2^), fat mass (22.2 vs. 19.6 kg), body cell mass (23.0 vs. 20.6 kg), waist circumference (83.8 vs. 83.0 cm), abdomen circumference (89.0 vs. 88.9 cm), and hip circumference (96.7 vs. 98.7 cm). Both young and older women did not significantly differ in sleep–wake timing, level of hs-CRP and level of vitamin D on the study day.

**Table 1:** General characteristics of the study participants

Character	Young women	Older women	Group comparison
	(*n* = 20)	(*n* = 20)	
	Mean ± SD	Mean ± SD	*P* value
General characteristics			
Weight (kg)	67.11 ± 10.61	68.45 ± 8.69	0.665
Height (m)	1.68 ± 0.05	1.70 ± 0.06	0.364
BMI (kg/m^2^)	23.65 ± 2.95	23.72 ± 2.51	0.936
Fat mass (kg)	21.04 ± 6.56	21.28 ± 6.27	0.906
Body cell mass (kg)	24.49 ± 2.92	22.20 ± 2.48	**0.011**
Waist circumference (cm)	72.90 ± 7.99	83.55 ± 9.44	**< 0.001**
Abdomen circumference (cm)	80.70 ± 9.04	88.95 ± 9.75	**0.005**
Hip circumference (cm)	92.05 ± 9.29	97.40 ± 8.26	0.062
Sleep quality (score)	5.90 ± 1.97	5.85 ± 2.50	0.944
Daytime sleepiness (score)	6.45 ± 3.17	6.00 ± 3.23	0.659
ME (score)	53.70 ± 11.73	58.90 ± 11.69	0.168
Study day			
hs-CRP (mg/l)	1.39 ± 0.94	1.60 ± 1.11	0.525
Vitamin D (ng/ml)	36.07 ± 19.93	40.11 ± 16.53	0.490
Awakening time (hh:mm) ^a^	06:14 ± 24	06:34 ± 19	0.154
Sleep onset time (hh:mm)^a^	23:10 ± 52	23:03 ± 74	0.709

BMI, body mass index; ME, morningness-eveningness; SD, standard deviation

^a^ Standard deviation is given in minutes

Significant differences between age groups are shown in bold

### Age group comparisons concerning 24-h variation of OGG1, oxidative DNA/RNA damage, TNF-α, cortisol, and CBT

**Fig. 1:**
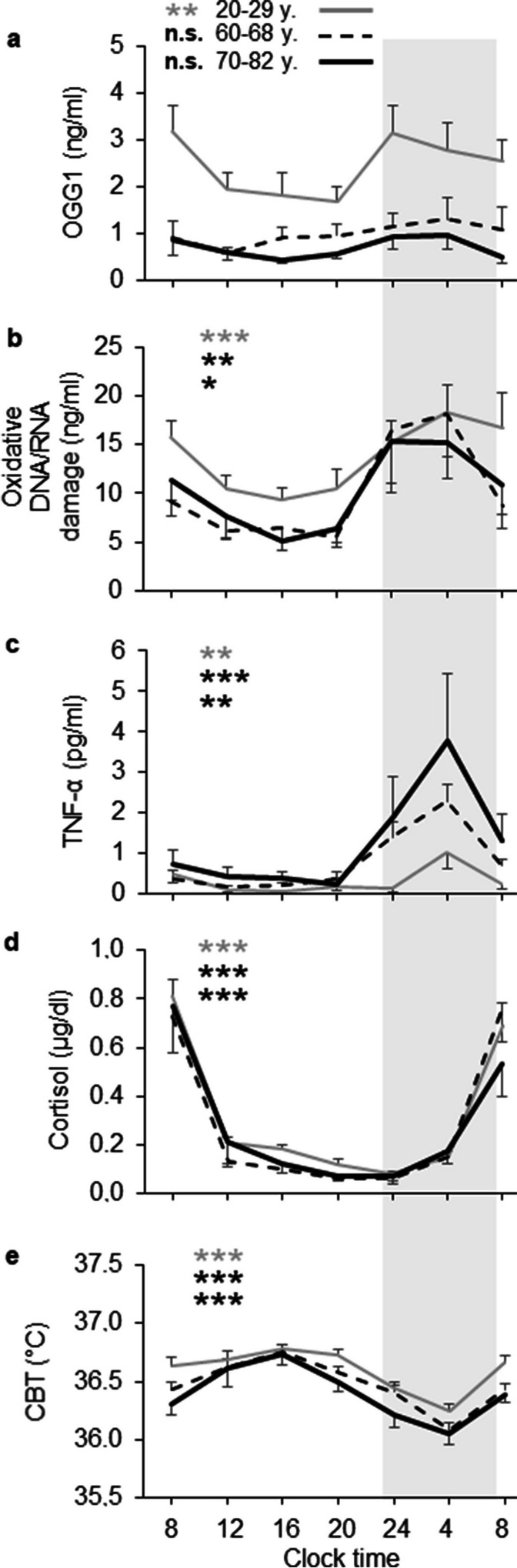
The 24-h profiles for salivary levels of (**a**) OGG1, (**b**) oxidative DNA/RNA damage, (**c**) TNF-α, (**d**) cortisol and (**e**) core body temperature (CBT) in 20 young women (grew lines) and 20 older women (black lines) for comparison. The older women were separated in women aged 60–68 years (dashed black lines, *N* = 13) and 70–82 years (solid black lines, *N* = 7). Salivary samples were collected in 4-h intervals at seven time-points (8, 12, 16, 20, 24, 4, and 8 h). All data represent mean values and standard errors of the mean (shown as half bars). The gray shaded area represents mean sleep time. Asterisks indicate significance of 24-h variation (Friedman tests), ** *P* < 0.010; *** *P* < 0.001; n.s. not significant

Salivary protein levels of OGG1 exhibited a significant 24-h variation in young women, but not in older women (young women: χ^2^ = 21.7, *P* = 0.001; older women: χ^2^ = 11.7, *P* = 0.070; Friedman tests). In both age groups, salivary levels of oxidative DNA/RNA damage, TNF-α and cortisol varied significantly within 24 h (young women: χ^2^ = 26.4, *P* < 0.001 for oxidative damage; χ^2^ = 17.9, *P* = 0.006 for TNF-α; χ^2^ = 89.3, *P* < 0.001 for cortisol; older women: χ^2^ = 34.4, *P* < 0.001 for oxidative damage; χ^2^ = 55.4, *P* < 0.001 for TNF-α; χ^2^ = 89.5, *P* < 0.001 for cortisol). CBT also demonstrated significant 24-h variation in both age groups (young women: χ^2^ = 58.9, *P* < 0.001; older women: χ^2^ = 82.6, *P* < 0.001). Since the age range of the older group was large (60–82 years), additional analyses were performed by splitting the older group into two subgroups, aged 60–68 years and 70–82 years. As well as the whole older group, both older subgroups showed no significant 24-h variation for OGG1 levels, but significant 24-h variation for the other diurnal traits (Fig. 1).

Table 2 displays for each circadian parameter the mean day level and mean night level, corresponding to the waking time and sleeping time of the women. The levels for OGG1 did not significantly differ between day and night, neither in young nor in older women. In contrast, both age groups exhibited significantly lower day levels than night levels for oxidative DNA/RNA damage and TNF-α (Fig. 1). The reverse was observed for cortisol and CBT, where the day levels were significantly higher than the night levels. With respect to the older women, only women aged 60–68 years showed significant different cortisol levels between day and night, while women aged 70–82 years did not. Mixed model analyses found no interaction between time (day vs. night level) and age (young vs. older women) for OGG1 (*P* = 0.609), TNF-α (*P* = 0.072), cortisol (*P* = 0.482) and CBT (*P* = 0.773), while a significant interaction occurred for oxidative DNA/RNA damage (*P* = 0.002).

**Table 2:** Comparison of the mean day and night levels for OGG1, oxidative RNA/DNA damage, TNF-α, cortisol and core body temperature

Parameter	Day level ^a^		Night level ^a^		Time comparison
	Mean	SEM	Mean	SEM	*P* value
Young women (*n* = 20)					
OGG1 (ng/ml)	2.24	0.26	2.96	0.56	0.117
Oxid. DNA/RNA damage (ng/ml)	12.55	1.21	36.34	0.06	** < 0.001**
TNF-α (pg/ml)	0.21	0.07	0.58	0.21	**0.011**
Cortisol (µg/dl)	0.40	0.03	0.12	0.02	** < 0.001**
CBT (°C)	36.70	0.05	36.34	0.06	** < 0.001**
Older women, 60–68 y (*n* = 13)					
OGG1 (ng/ml)	0.90	0.18	1.23	0.35	0.311
Oxid. DNA/RNA damage (ng/ml)	7.24	1.00	36.25	0.04	** < 0.001**
TNF-α (pg/ml)	0.37	0.08	1.84	0.34	** < 0.001**
Cortisol (µg/dl)	0.36	0.06	0.10	0.02	**0.001**
CBT (°C)	36.56	0.04	36.25	0.04	** < 0.001**
Older women, 70–82 y (*n* = 7)					
OGG1 (ng/ml)	0.63	0.09	1.08	0.26	0.237
Oxid. DNA/RNA damage (ng/ml)	8.25	1.62	36.13	0.09	**0.018**
TNF-α (pg/ml)	0.63	0.21	3.26	1.02	**0.018**
Cortisol (µg/dl)	0.34	0.07	0.12	0.03	0.063
CBT (°C)	36.51	0.08	36.13	0.09	**0.018**

CBT, core body temperature; SEM, standard error of the mean

^a^ The day level corresponds to the waking time and the night level to the sleeping time of the women

Significant difference between day level and night level is shown in bold

The highest levels of OGG1 appeared in older women at 04:00 h and in young women at 24:00 h. The highest levels of oxidative DNA/RNA damage and TNF-α occurred in both age groups at 04:00 h. A separate analysis of the older subgroups revealed that the maximum level for TNF-α at 04:00 h was much higher in the oldest group (70–82 years) than in the group aged 60–68 years (3.79 pg/ml vs. 2.28 pg/ml), but differences were not significant. Both young and older women reached highest cortisol levels after awakening at 08:00 h in the morning, followed by a strong decline until 12:00 h, subsequently a slight decline until minimum levels at 20:00 h in older women and at 24:00 h in young women. The lowest levels of cortisol thus appeared 4 h earlier in older women compared to young women. The 24-h profiles for cortisol were similar in young and older women.

The 24-h mean level for OGG1 was strongly and significantly lowered in older women compared to young women (0.90 ng/ml vs. 2.41 ng/ml, *P* < 0.00001). At each time-point of the 24-h period, older women exhibited significantly lower levels of OGG1 than young women did (08:00 h: *P* < 0.001; 12:00 h: *P* < 0.001; 16:00 h: *P* = 0.007; 20:00 h: *P* = 0.015; 24:00 h: *P* < 0.001; 04:00 h: *P* = 0.001; 08:00 h: *P* < 0.001). Unlike OGG1, the 24-h mean level for TNF-α was significantly and strongly raised in the older women relative to the young women (0.91 ng/ml vs. 0.43 ng/ml, *P* = 0.011). Older women demonstrated at each time-point of the 24-h period higher protein levels of TNF-α than young women; the age group differences were significant at 16:00 h, 24:00 h, 04:00 h and 08:00 h (16:00 h: *P* = 0.009; 24:00 h: *P* < 0.001; 04:00 h: *P* < 0.001; 08:00 h: *P* = 0.003). The 24-h mean levels for oxidative DNA/RNA damage and cortisol did not differ between age groups. A separate comparison of 24-h mean levels between the older subgroups demonstrated a lower 24-h mean level for OGG1 (0.70 ng/ml vs. 0.99 ng/ml) and a higher 24-h mean level for TNF-α (1.25 pg/ml vs. 0.78 pg/ml) in the very old group (70–82 years) compared to the group aged 60–68 years, although differences were not statistically significant. In contrast, the 24-h mean levels for oxidative DNA/RNA damage (10.25 ng/ml vs. 10.09 ng/ml) and cortisol (0.28 µg/dl vs. 0.28 µg/dl) were similar in both older subgroups.

The 24-h mean for CBT was slightly and significantly lower in older women compared to young women (36.45 °C vs. 36.59, *P* = 0.022). The diurnal amplitude (% mean) for CBT did not differ between older and young women (2.09% mean vs. 1.93% mean, *P* = 0.397).

### Temporal relationships of OGG1 with oxidative DNA/RNA damage, TNF-α and cortisol

In both age groups, the protein levels of OGG1 exhibited similar 24-h variation as levels of oxidative DNA/RNA damage, but both characters showed mainly weak and non-significant correlations.

**Fig. 2:**
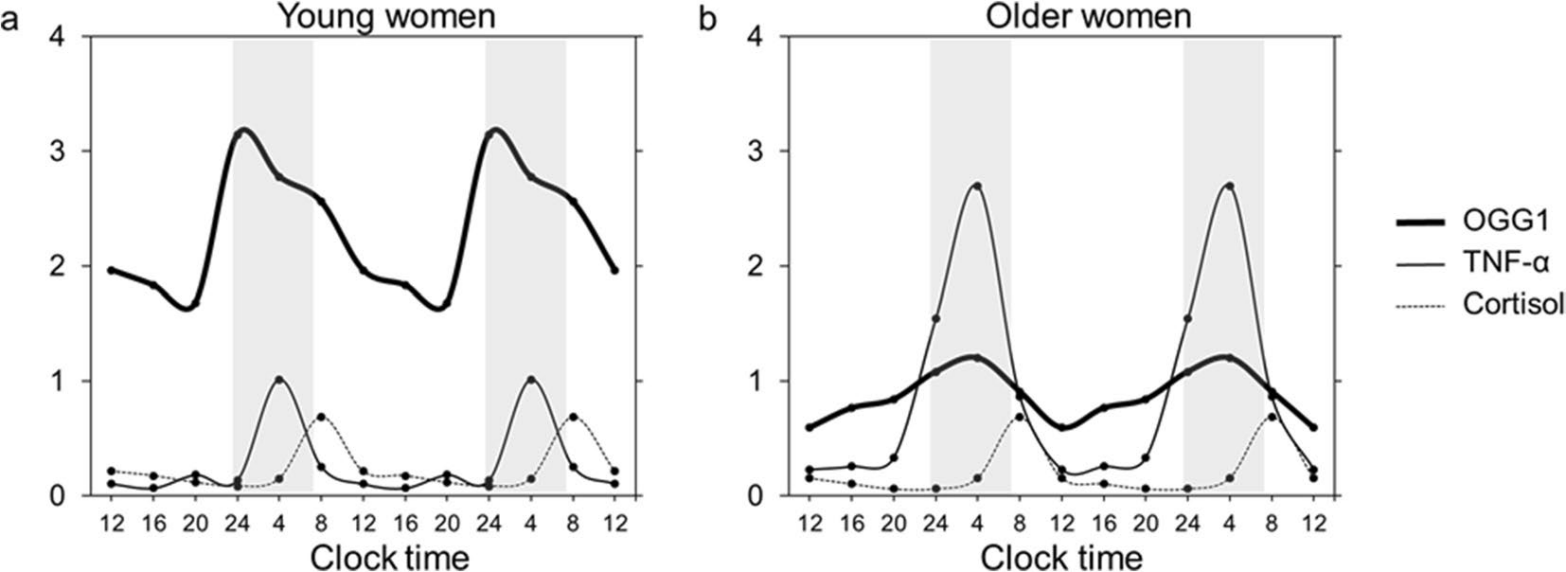
Temporal relationships between the 24-h profiles for salivary levels of OGG1, TNF-α and cortisol in (**a**) 20 young women and (**b**) 20 older women for comparison. Mean values are given as double-plots, provided as spline curves, to show rhythmicity. The gray-shaded area represents mean sleeping time

The curve for OGG1 of young women was advanced by four hours relative to the curve for TNF-α, reaching highest levels at 24:00 h (Fig. 2). The curves for OGG1 and TNF-α of older women were in phase exhibiting highest levels at equal time-points (04:00 h). There were negative and significant correlations between OGG1 levels at later daytime (12:00 h, 16:00 h, 20:00 h) and TNF-α levels at early daytime (04:00 h, 08:00 h) in young women (rs = -0.49 to rs = -0.59, *P* < 0.050 to *P* < 0.010), but not in older women.

The curve for OGG1 was phase-advanced against the curve of cortisol by 4 h in older women and by 8 h in young women. Positive and significant correlations between the levels of OGG1 and cortisol were observed especially at 12:00 h, 20:00 h and 24:00 h in young women (rs = 0.56, 0.54, 0.63; *P* = 0.011, 0.014, 0.003). Older women showed less significant relationships, except for a positive correlation between the OGG1 levels at 20:00 h and cortisol levels at 08:00 h (rs = 0.63, *P* = 0.003).

In both age groups, the curve for TNF-α was time-shifted against the curve for cortisol; highest levels of TNF-α occurred four hours earlier (04:00 h) than highest levels of cortisol (08:00 h). Between 04:00 h and 08:00 h, the TNF-α level strongly decreased, while the cortisol level strongly increased. There were negative and significant correlations between cortisol levels at 04:00 h and TNF-α levels at 08:00 h (rs = -0.53, *P* = 0.017) as well as between cortisol levels at 12:00 h and TNF-α levels at 08:00 h the day after (rs = -0.67, *P* = 0.001) in young women, but not in older women.

### Predictors of the 24-h mean level of OGG1

**Fig. 3:**
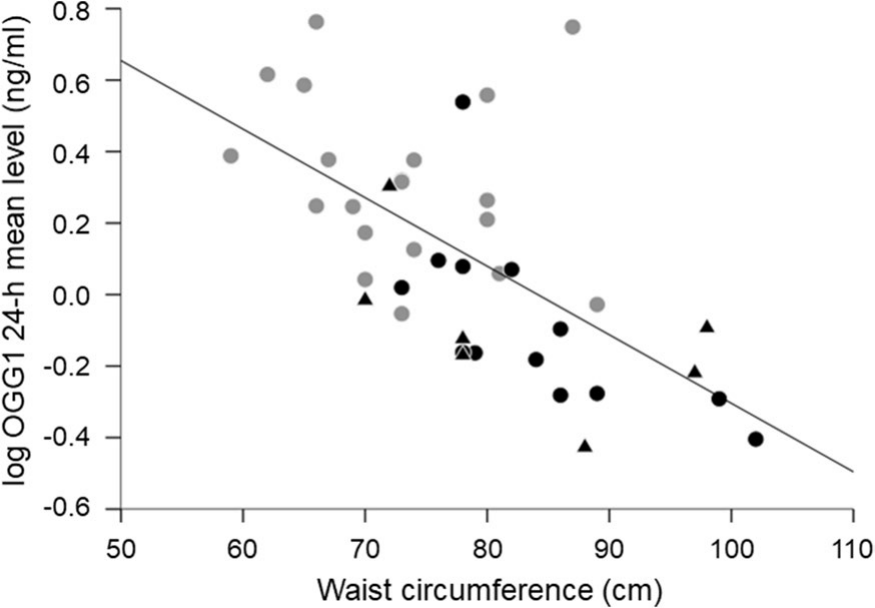
Correlation between 24-h mean level of OGG1 and waist circumference (*r* = -0.627, *P* = 0.00001) in healthy women (*N* = 40; young women: grew circles; older women 60–68 years: black circles; older women 70–82 years: black triangles)

Regressions analysis detected, that age, the 24-h mean level of TNF-α and waist circumference were negative and significant predictors of the 24-h mean level of OGG1, explaining together 56% of variance of OGG1 (*R*^2^ = 0.61, adjusted *R*^2^ = 0.56; *F* = 10.74, *P* < 0.00001, Table 3). In contrast, the 24-h mean levels of oxidative DNA/RNA damage and cortisol were no significant predictors of OGG1. Figure 3 displays the negative relationship between OGG1 and waist circumference. The older women had significantly higher waist circumference than young women, although no differences in BMI were observed between age groups. The older subgroups (60–68 years and 70–82 years), who are indicated by different signs in the figure, were equally distributed along the regression line.

**Table 3:** Regression analysis showing relationship between the 24-h mean protein level of OGG1 (dependent variable) with 24-h mean levels of TNF-α, oxidative RNA/DNA damage and cortisol as well as age and waist circumference in 40 healthy women (*F* = 10.74, *P* < 0.00001, adjusted *R*^2^ = 0.56)

Independent variables	B	*P* value
Age (years)	-0.005	**0.023**
Waist circumference (cm)	-0.012	**0.003**
TNF-α (pg/ml)^a^	-0.151	**0.039**
Oxidative DNA/RNA damage (ng/ml) ^a^	-0.104	0.384
Cortisol (µg/dl)^a^	0.142	0.411

B, regression coefficient

^a^ Log-transformed values were used

Significant predictors of OGG1 level are shown in bold

## Discussion

### Age-related differences in 24-h profiles for OGG1, TNF-α and cortisol and CBT

For the first time, the present study provided 24-h-profiles for protein levels of OGG1 in healthy older humans. The older women exhibited throughout the whole 24-h period strongly and significantly lowered salivary protein levels of OGG1 than young women. The lowered levels of OGG1 in the older women are in line with results of human and animal studies, which analyzed OGG1 levels at a single time-point. Picca and colleagues [27] noted lower protein levels of OGG1 in muscles of older adults, compared to young adults. Chen and colleagues [28] reported a significant age-dependent decrease of OGG1 activity in vitro in human blood lymphocytes. Animal results demonstrated a decrease in protein and mRNA levels of OGG1 with age [5]. The present findings in healthy women suggest a strong decrease of protein levels of OGG1 during healthy cellular aging. The lowered enzyme levels of OGG1 may affect repair of oxidative DNA damage during aging, because results of Imam and colleagues [29] demonstrated in animals a significant age-dependent decrease in incision activities of OGG1. Future studies are needed to clarify the underlying age-related mechanisms.

A previous study demonstrated a 24-h rhythmicity for gene expression and enzymatic activity of OGG1 in blood lymphocytes of young adults [6]. We confirmed for protein levels of OGG1 a highly significant 24-h variation in saliva of young women. In contrast to young women, we found no significant 24-h variation of protein levels of OGG1 in older women. This is attributable to their lowered protein levels of OGG1. Additional studies should explore whether the absence of 24-h variation may impair the maintenance of cellular homeostasis in DNA repair in the aged.

The present study found a significant 24-h variation of protein levels for TNF-α in the saliva of both young and older women. Highest levels of TNF-α occurred consistently in both age groups at 04:00 h. This timing agrees with previous results in young male adult dayworkers who reached highest TNF-α levels in blood at 04:00 h [30]. We found higher TNF-α levels in older women than in young women throughout the whole 24-h period; age group differences were significant at four of seven time-points. The age group differences were not attributable to differences in vitamin D levels [24], which were similar in older and young women. The increased TNF-α levels in the older women are in line with studies reporting a general increase in TNF-α levels during aging [31]. Thus, the raised levels of TNF-α in the healthy older women agree with the concept of inflammaging, that healthy aging is accompanied by a chronic low-grade inflammation, even in the absence of diseases [11, 12].

Previous studies analyzed 24-h profiles for cortisol levels in blood plasma of older humans [32–34] and in saliva of frailty older humans [35]. This study provided 24-h profiles for salivary levels of cortisol in healthy older women. We observed a significant 24-h variation in older women and their 24-h profiles were similar to those of young women. Consistent with a previous study in young adults in blood, the present study found in saliva of older and young women highest levels of cortisol in the morning after awakening around 08:00 h and lowest levels between 20:00 h and 24:00 h [6]. In line with another human study, which analyzed cortisol levels in blood plasma [34], we observed in saliva, that the minimum levels of cortisol appeared in older women about four hours earlier than in young women, indicating a time shift by four hours between age groups. This time shift was not attributable to sleep–wake times, because bedtimes did not differ between age groups in the present study.

In the present study, the 24-h amplitude for the CBT rhythm did not differ between the older and young women. This agrees with results of Monk and colleagues [36] who found no evidence that older subjects had generally lower temperature rhythm amplitudes than younger adults. Although some earlier studies observed a decrease of amplitude of CBT in older men [37], this was not consistently found in older women. The absence of a decreased amplitude in the older women of the present study may be due to the fact that the older group was healthy and did not differ in their sleep quality from the young women. Thus, pathological processes as well as sleep disorders did not affect the rhythm of temperature regulation in the older women.

### Relationship of the 24-h profile of OGG1 with profiles of oxidative DNA/RNA damage, TNF-α and cortisol

The salivary protein levels of OGG1 correlated over the 24-h period in both age groups weakly and mostly non-significantly with levels of oxidative DNA/RNA damage. This agrees with studies reporting that the expression of the *Ogg1* gene is not modulated in response to DNA damage [38], but more likely exhibits a circadian variation that is regulated by the endogenous biological clock [6].

The present study compared for the first time 24-h protein levels of OGG1 and TNF-α in healthy humans. The 24-h curve for OGG1 of young women was phase-advanced by four hours against the curve of TNF-α. This was not observed in the older women displaying both curves in phase. One explanation could be the raised TNF-α levels in the older women. It is known that TNF-α modulates the functioning of the central biological clock, which is located within the hypothalamic suprachiasmatic nuclei (SCN) [39]. TNF-α also modifies the expression of clock genes, which are involved in the generation of 24-h rhythms [40]. A previous study showed that a deregulated molecular biological clock resulted in an abolishment of circadian variation of *Ogg1* gene expression [6]. This may explain the reduced levels of OGG1 together with the increased levels of TNF-α in the older women.

This study firstly compared the 24-h time courses for protein levels of OGG1 and cortisol in saliva. In both age groups, the curve for OGG1 was phase-advanced against the curve of cortisol. The young women demonstrated positive and significant correlations between protein levels of OGG1 and cortisol. This is consistent with previous findings reporting a positive correlation between *Ogg1* gene expression and plasma cortisol concentration in lymphocytes of healthy young adults [6]. The positive relationship is in line with in vitro results of O’Brian and colleagues [41] that cortisol concentrations < 10 µg/dl increased DNA repair capacity. Contrary to young women, the older women showed less significant relationships between OGG1 and cortisol. This could be explained by a diminished cellular responsiveness to glucocorticoids in older age [42].

Salivary protein levels of TNF-α and cortisol were compared over the course of a 24-h day. In both age groups, the curve for TNF-α was phase-advanced by 4 h against the curve for cortisol, reaching highest levels at 04:00 and 08:00 h, respectively. Higher cortisol levels at 04:00 h were significantly related to lower TNF-α levels at 08:00 h in young women, but weaker and non-significant in older women. The negative and significant relationship in young women is in line with a previous study, in which patients with cardiovascular diseases with lower cortisol levels upon waking showed higher levels of TNF-α [43]. This time relationship can be explained by the anti-inflammatory effect of the hormone cortisol, which downregulates the inflammatory activities of the pro-inflammatory cytokine TNF-α [44]. The authors showed that both a physiological dose of hydrocortisone as well as stress-induced levels of glucocorticoids suppressed the production of TNF-α in human blood plasma. The non-significant relationship, that we observed in older women, could be explained by a lower glucocorticoid sensitivity of pro-inflammatory cytokines, which has been reported for older men compared to young men [45].

### TNF-α and waist circumference as predictors of OGG1

This study found that age, 24-h mean level of the inflammation marker TNF-α and waist circumference were negative predictors of the 24-h mean level of OGG1, while oxidative DNA/RNA damage and cortisol were no significant predictors. The negative relationship between OGG1 and TNF-α is in line with previous results demonstrating that OGG1 might contribute to the downregulation of the TNF-α level [46]. Moreover, a recent study showed that mitochondrial OGG1 confers a protective role in age-associated inflammation [47]. In addition, elevated levels of mitochondrial OGG1 can reverse aging-associated inflammation in transgenic mice [47].

There might be a direct role of TNF-α on OGG1. This is supported by a mechanistic cellular study showing that exposure to physiologically relevant levels of TNF-α inactivates a common OGG1 allelic variant (rs1052133, S326C) in mammalian cells [14]. This variant predominates in 40–60% of Asian and in 25–40% of Caucasian populations [48]. However, there might be additional mechanisms. Future mechanistic studies should therefore test whether there is an epigenetic repression of OGG1 by TNF-α.

Inflammation is associated with higher central body fat mass [10]. We found a negative association between the protein level of OGG1 and waist circumference, which is indicative of central body fat. This demonstrates that women with higher waist circumference had lower protein levels of OGG1 and vice versa. This finding is in line with animal results reporting increased adiposity in OGG1-deficient mice [49]. It can be explained by a recent finding that OGG1 alters energetics in adipose tissue and higher levels of OGG1 protect against obesity [50].

Inflammaging occurs in aged adipose tissue, where it contributes to adipocyte hypertrophy and dysfunction; there is also an increased infiltration of immune cells in adipose tissue and these immune cells secreted proinflammatory cytokines [51]. Animal studies showed that adipose-tissue derived TNF-α (AT-TNF) activity was elevated in older animals, while AT-TNF protein levels where higher in young animals, suggesting that they may secrete an inhibitor that reduces AT-TNF activity [52]. A high fat diet elevated the AT-TNF activity in animals [53]. Moreover, the circulating levels of TNF-α can be nutritionally regulated and they decrease after weight loss in obese humans [54]. Future studies should explore the effect of diet on AT-TNF in older and young humans.

The circadian variation in circulating TNF-α originates from a combined influence of the circadian system and sleep [55]. The circadian oscillation of TNF-α gene expression is regulated by a molecular clock, generated by intracellular feedback loops of core clock genes and proteins [56]. The clock protein CLOCK acetylates a subunit of NF-kB to induce suppression of TNF and its rhythmic sequestration by the clock protein BMAL1 can drive oscillations in TNF expression [57]. In addition, the rhythm of TNF-α is dependent on sleep. TNF-α peaks in healthy humans during nighttime, is classified as somnogenic and is involved in sleep regulation [58]. The difference between the TNF-α peak levels of older and younger women in the present study was not due to differences in sleep, because both age groups did not differ in their bedtimes, sleep quality and daytime sleepiness.

### Limitations of the study

The present findings are limited by some factors. A first limitation pertains to the observational nature of the present study and the lack of mechanistic data that support the present results. Future experimental and interventional studies are needed to get more insight into mechanisms related to how TNF-α can suppress OGG1 expression, for example by testing in vitro epigenetic repression. A second limitation refers to consider only TNF-α as a biomarker for inflammaging. Since TNF-α alone is not representative of the whole inflammaging, a panel of other cytokines, including IL-6 and IL1b, should be additionally analyzed to get a deeper insight into the complex way of inflammaging. A third limitation of this study is that the diet consumed by the participants and the effect of diet on TNF-α levels was not analyzed. Animal studies showed that high fat diet elevates adipose-tissue derived TNF-α (AT-TNF) activity [53] and that AT-TNF activity is elevated in older animals. Moreover, AT-TNF-α activity increased in mature animals in relation to adipose cell size [52]. In humans, a Mediterranean diet (olive oil, fiber, fruit or vegetables) as well as the consumption of olive oil as a single item were inversely and significantly associated with TNF-α in coronary venous blood [58]. Therefore, further research is necessary to compare the effects of dietary components as well as the effects of low and high fat diet on adipose tissue-derived TNF-α in older and young humans. A fourth limitation concerns the restriction to female participants that limits the generalizability of the study. However, Chen and colleagues [28] reported absence of sex differences in human OGG1 repair capacity. Nevertheless, additional studies may extend the investigation on male participants in order to find out whether women and men differ in their relationship of OGG1 with TNF-α.

## Conclusions

This study examined in humans the 24-h variation of salivary protein levels of OGG1 and its relationship with other characters. The first main finding was a strong age-related decrease of protein levels of OGG1 throughout the whole 24-h period and a loss of 24-h variation during healthy aging. Whether the absence of 24-h variation impairs the maintenance of cellular homeostasis in the aged should investigate future studies. The second main finding of this study was that TNF-α and waist circumference were negative predictors of OGG1. This provides evidence that proinflammatory processes may be related to DNA repair. The result that the 24-h mean level of TNF-α was more than twice as high in healthy older relative to young women, confirms the concept of inflammaging.

## References

[1] Barzilai A, Yamamoto K (2004). DNA damage responses to oxidative stress. DNA Repair (Amst).

[2] Cadet J, Douki T, Ravanat JL (2006). One-electron oxidation of DNA and inflammation processes. Nat Chem Biol.

[3] Monden Y, Arai T, Asano M, Ohtsuka E, Aburatani H, Nishimura S (1999). Human MMH (OGG1) type 1a protein is a major enzyme for repair of 8-hydroxyguanine lesions in human cells. Biochem Biophys Res Commun.

[4] Radicella JP, Dherin C, Desmaze C, Fox MS, Boiteux S (1997). Cloning and characterization of hOGG1, a human homolog of the OGG1 gene of Saccharomyces cerevisiae. Proc Natl Acad Sci U S A.

[5] Tian F, Tong TJ, Zhang ZY, McNutt MA, Liu XW (2009). Age-dependent down-regulation of mitochondrial 8-oxoguanine DNA glycosylase in SAM-P/8 mouse brain and its effect on brain aging. Rejuvenation Res.

[6] Manzella N, Bracci M, Strafella E, Staffolani S, Ciarapica V, Copertaro A, Rapisarda V, Ledda C, Amati M, Valentino M, Tomasetti M, Stevens RG, Santarelli L (2015). Circadian Modulation of 8-Oxoguanine DNA Damage Repair. Sci Rep.

[7] Duffy JF, Zitting KM, Chinoy ED (2015). Aging and Circadian Rhythms. Sleep Med Clin.

[8] Turrens JF (2003). Mitochondrial formation of reactive oxygen species. J Physiol.

[9] Mittal M, Siddiqui MR, Tran K, Reddy SP, Malik AB (2014). Reactive oxygen species in inflammation and tissue injury. Antioxid Redox Signal.

[10] Arbel Y, Birati EY, Shapira I, Finn T, Berliner S, Rogowski O. Comparison of different anthropometric measurements and inflammatory biomarkers. Int J Inflam. 2012;124693. 10.1155/2012/124693. 10.1155/2012/124693 PMC3362833 22675656

[11] Gerli R, Monti D, Bistoni O, Mazzone AM, Peri G, Cossarizza A, Di Gioacchino M, Cesarotti ME, Doni A, Mantovani A, Franceschi C, Paganelli R (2000). Chemokines, sTNF-Rs and sCD30 serum levels in healthy aged people and centenarians. Mech Ageing Dev.

[12] Franceschi C, Bonafè M, Valensin S, Olivieri F, De Luca M, Ottaviani E, De Benedictis G (2000). Inflamm-aging: An evolutionary perspective on immunosenescence. Ann N Y Acad Sci..

[13] Cain DW, Cidlowski JA (2017). Immune regulation by glucocorticoids. Nat Rev Immunol.

[14] Morreall J, Limpose K, Sheppard C, Kow YW, Werner E, Doetsch PW (2015). Inactivation of a common OGG1 variant by TNF-alpha in mammalian cells. DNA Repair (Amst).

[15] Cavadini G, Petrzilka S, Kohler P, Jud C, Tobler I, Birchler T, Fontana A (2007). TNF-alpha suppresses the expression of clock genes by interfering with E-box-mediated transcription. Proc Natl Acad Sci U S A.

[16] Veglia F, Cighetti G, De Franceschi M, Zingaro L, Boccotti L, Tremoli E, Cavalca V (2006). Age- and gender-related oxidative status determined in healthy subjects by means of OXY-SCORE, a potential new comprehensive index. Biomarkers.

[17] Aomatsu M, Kato T, Kasahara E, Kitagawa S (2013). Gender difference in tumor necrosis factor-α production in human neutrophils stimulated by lipopolysaccharide and interferon-γ. Biochem Biophys Res Commun.

[18] Sofer Y, Osher E, Limor R, Shefer G, Marcus Y, Shapira I, Tordjman K, Greenman Y, Berliner S, Stern N (2016). Gender determines serum free cortisol: higher levels in men. Endocr Pract.

[19] Lewy AJ, Wehr TA, Goodwin FK, Newsome DA, Markey SP (1980). Light suppresses melatonin secretion in humans. Science.

[20] Horne JA, Östberg O (1976). A self-assessment questionnaire to determine morningness-eveningness in human circadian rhythms. Int J Chronobiol.

[21] Griefahn B, Künemund C, Bröde P, Mehnert P (2001). Zur Validität der deutschen Übersetzung des Morningness-Eveningness-Questionnaires von Horne und Östberg. Somnologie.

[22] Buysse DJ, Reynolds CF, Monk TH, Berman SR, Kupfer DJ (1989). The Pittsburgh sleep quality index: a new instrument for psychiatric practice and research. Psychiatry Res.

[23] Johns MW (1991). A new method for measuring daytime sleepiness: the Epworth sleepiness scale. Sleep.

[24] Ian N, Luo G, Yang X, Cheng Y, Zhang Y, Wang X, Wang X, Xie T, Li G, Liu Z, Zhong N (2014). 25-Hydroxyvitamin D3-deficiency enhances oxidative stress and corticosteroid resistance in severe asthma exacerbation. PLoS One.

[25] Snedecor GW, Cochran WG. Statistical methods. 7th ed. Ames, USA: Iowa State University Press; 1980.

[26] Goyal M, Goel A, Kumar P, Bajpai M, Verma NS, Kant S, Tiwari S (2008). Circadian rhythm of peak expiratory flow rate in healthy north Indian men. Indian J Physiol Pharmacol.

[27] Picca A, Mankowski RT, Kamenov G, Anton SD, Manini TM, Buford TW, Saini SK, Calvani R, Landi F, Bernabei R, Marzetti E, Leeuwenburgh C (2019). Advanced age is associated with iron dyshomeostasis and mitochondrial DNA damage in human skeletal muscle. Cells.

[28] Chen SK, Hsieh WA, Tsai MH, Chen CC, Hong AI, Wei YH, Chang WP (2003). Age-associated decrease of oxidative repair enzymes, human 8-oxoguanine DNA glycosylases (hOgg1), in human aging. J Radiat Res.

[29] Imam SZ, Karahalil B, Hogue BA, Souza-Pinto NC, Bohr VA (2006). Mitochondrial and nuclear DNA-repair capacity of various brain regions in mouse is altered in an age-dependent manner. Neurobiol Aging.

[30] Crispim CA, Padilha HG, Zimberg IZ, Waterhouse J, Dattilo M, Tufik S, de Mello MT (2012). Adipokine levels are altered by shiftwork: a preliminary study. Chronobiol Int.

[31] De Gonzalo-Calvo D, Neitzert K, Fernández M, Vega-Naredo I, Caballero B, García-Macía M, Suárez FM, Rodríguez-Colunga MJ, Solano JJ, Coto-Montes A (2010). Differential inflammatory responses in aging and disease: TNF-alpha and IL-6 as possible biomarkers. Free Radic Biol Med.

[32] Kalleinen N, Polo-Kantola P, Irjala K, Porkka-Heiskanen T, Vahlberg T, Virkki A, Polo O (2008). 24-hour serum levels of growth hormone, prolactin, and cortisol in pre- and postmenopausal women: the effect of combined estrogen and progestin treatment. J Clin Endocrinol Metab.

[33] Lupien S, Lecours AR, Schwartz G, Sharma S, Hauger RL, Meaney MJ, Nair NP (1996). Longitudinal study of basal cortisol levels in healthy elderly subjects: evidence for subgroups. Neurobiol Aging.

[34] Purnell JQ, Brandon DD, Isabelle LM, Loriaux DL, Samuels MH (2004). Association of 24-hour cortisol production rates, cortisol-binding globulin, and plasma-free cortisol levels with body composition, leptin levels, and aging in adult men and women. J Clin Endocrinol Metab.

[35] Varadhan R, Walston J, Cappola AR, Carlson MC, Wand GS, Fried LP (2008). Higher levels and blunted diurnal variation of cortisol in frail older women. J Gerontol A Biol Sci Med Sci.

[36] Monk TH, Buysse DJ, Reynolds CF, Kupfer DJ, Houck PR (1995). Circadian temperature rhythms of older people. Exp Gerontol.

[37] Vitiello MV, Smallwood RG, Avery DH, Pascualy RA, Martin DC, Prinz PN (1986). Circadian temperature rhythms in young adult and aged men. Neurobiol Aging.

[38] Mistry P, Herbert KE (2003). Modulation of hOGG1 DNA repair enzyme in human cultured cells in response to pro-oxidant and antioxidant challenge. Free Radic Biol Med.

[39] Lundkvist GB, Hill RH, Kristensson K (2002). Disruption of circadian rhythms in synaptic activity of the suprachiasmatic nuclei by African trypanosomes and cytokines. Neurobiol Dis.

[40] Lopez M, Meier D, Müller A, Franken P, Fujita J, Fontana A (2014). Tumor necrosis factor and transforming growth factor β regulate clock genes by controlling the expression of the cold inducible RNA-binding protein (CIRBP). J Biol Chem.

[41] O'Brien SN, Larcom LL, Baxley EG (1993). Correlates of plasma cortisol and DNA repair in human peripheral lymphocytes: suppression of repair in women taking estrogen. Horm Res.

[42] Revskoy S, Redei E (2000). Decreased in vitro sensitivity to dexamethasone in corticotropes from middle-age rats. Exp Gerontol.

[43] DeSantis AS, DiezRoux AV, Hajat A, Aiello AE, Golden SH, Jenny NS, Seeman TE, Shea S (2012). Associations of salivary cortisol levels with inflammatory markers: the multi-ethnic study of atherosclerosis. Psychoneuroendocrinology.

[44] DeRijk R, Michelson D, Karp B, Petrides J, Galliven E, Deuster P, Paciotti G, Gold PW, Sternberg EM (1997). Exercise and circadian rhythm-induced variations in plasma cortisol differentially regulate interleukin-1 beta (IL-1 beta), IL-6, and tumor necrosis factor-alpha (TNF alpha) production in humans: high sensitivity of TNF alpha and resistance of IL-6. J Clin Endocrinol Metab.

[45] Rohleder N, Kudielka BM, Hellhammer DH, Wolf JM, Kirschbaum C (2002). Age and sex steroid-related changes in glucocorticoid sensitivity of pro-inflammatory cytokine production after psychosocial stress. J Neuroimmunol.

[46] Dezor M, Dorszewska J, Florczak J, Kempisty B, Jaroszewska-Kolecka J, Rozycka A, Polrolniczak A, Bugaj R, Jagodzinski PP, Kozubski W (2011). Expression of 8-oxoguanine DNA glycosylase 1 (OGG1) and the level of p53 and TNF-αlpha proteins in peripheral lymphocytes of patients with Alzheimer's disease. Folia Neuropathol.

[47] Hussain M, Chu X, Duan Sahbaz B, Gray S, Pekhale K, Park JH, Croteau DL, Bohr VA (2023). Mitochondrial OGG1 expression reduces age-associated neuroinflammation by regulating cytosolic mitochondrial DNA. Free Radic Biol Med.

[48] Hung RJ, Hall J, Brennan P, Boffetta P (2005). Genetic polymorphisms in the base excision repair pathway and cancer risk: a HuGE review. Am J Epidemiol..

[49] Sampath H, Vartanian V, Rollins MR, Sakumi K, Nakabeppu Y, Lloyd RS (2012). 8-Oxoguanine DNA glycosylase (OGG1) deficiency increases susceptibility to obesity and metabolic dysfunction. PLoS One.

[50] Komakula SSB, Tumova J, Kumaraswamy D, Burchat N, Vartanian V, Ye H, Dobrzyn A, Lloyd RS, Sampath H (2018). The DNA repair protein OGG1 protects against obesity by altering mitochondrial energetics in white adipose tissue. Sci Rep.

[51] Zhang YX, Ou MY, Yang ZH, Sun Y, Li QF, Zhou SB (2023). Adipose tissue aging is regulated by an altered immune system. Front Immunol.

[52] Morin CL, Pagliassotti MJ, Windmiller D, Eckel RH (1997). Adipose tissue-derived tumor necrosis factor-alpha activity is elevated in older rats. J Gerontol A Biol Sci Med Sci.

[53] Morin CL, Eckel RH, Marcel T, Pagliassotti MJ (1997). High fat diets elevate adipose tissue-derived tumor necrosis factor-alpha activity. Endocrinology.

[54] Zahorska-Markiewicz B, Janowska J, Olszanecka-Glinianowicz M, Zurakowski A (2000). Serum concentrations of TNF-alpha and soluble TNF-alpha receptors in obesity. Int J Obes Relat Metab Disord.

[55] Lange T, Dimitrov S, Born J (2010). Effects of sleep and circadian rhythm on the human immune system. Ann N Y Acad Sci.

[56] Onoue T, Nishi G, Hikima JI, Sakai M, Kono T (2019). Circadian oscillation of TNF-α gene expression regulated by clock gene, BMAL1 and CLOCK1, in the Japanese medaka (Oryzias latipes). Int Immunopharmacol.

[57] Man K, Loudon A, Chawla A (2016). Immunity around the clock. Science.

[58] Serrano-Martinez M, Palacios M, Martinez-Losa E, Lezaun R, Maravi C, Prado M, Martínez JA, Martinez-Gonzalez MA (2005). A Mediterranean dietary style influences TNF-alpha and VCAM-1 coronary blood levels in unstable angina patients. Eur J Nutr.

